# CuAAC-inspired synthesis of 1,2,3-triazole-bridged porphyrin conjugates: an overview

**DOI:** 10.3762/bjoc.19.29

**Published:** 2023-03-22

**Authors:** Dileep Kumar Singh

**Affiliations:** 1 Department of Chemistry, Bipin Bihari College, Affiliated to Bundelkhand University, Jhansi, Uttar Pradesh, 284001, India

**Keywords:** azide–alkyne, click chemistry, CuAAC, 1,3-dipolar cycloaddition, porphyrin, 1,2,3-triazole

## Abstract

Among all the available approaches in organic synthesis, the “click chemistry” protocol is very common nowadays to covalently connect two diverse moieties in a single framework. Therefore, this review focuses on the synthesis and photophysical studies of β- and *meso*-substituted and 1,2,3-triazole-fused porphyrin conjugates. All of the porphyrin conjugates discussed here are synthesized via a copper(I)-catalyzed Huisgen 1,3-dipolar cycloaddition reaction between an azide and a terminal alkyne, also popular as "click reaction" or CuAAC reaction. Moreover, the 1,2,3-triazole ring also serves as a spacer and an electron transfer bridge between the porphyrin and the attached chromophores. In order to provide a critical overview of the synthesis and properties of various porphyrin-triazole hybrids, this review will discuss some of the key reactions involved in the preparation of triazole-linked porphyrin conjugates.

## Introduction

Porphyrins are an aromatic, immensely conjugated, and colorful macrocyclic array with remarkable optical, electrochemical, and biological features. It is one of the most fascinating heterocyclic molecules which have various applications in catalysis, materials sciences, photochemistry and medicinal chemistry. Owing to the strong absorption in the visible region, these 18π-electron systems also demonstrate excellent photoluminescence properties. Therefore, porphyrin hybrids have been widely used as efficient sensitizers for PDT applications and dye-sensitized solar cells (DSSCs). Because the majority of porphyrin hybrids are synthesized by modifying β- and *meso*-positions of porphyrin, thus numerous chromophoric groups and biologically important scaffolds have been attached to porphyrins at these positions in recent years.

**Figure 1:**
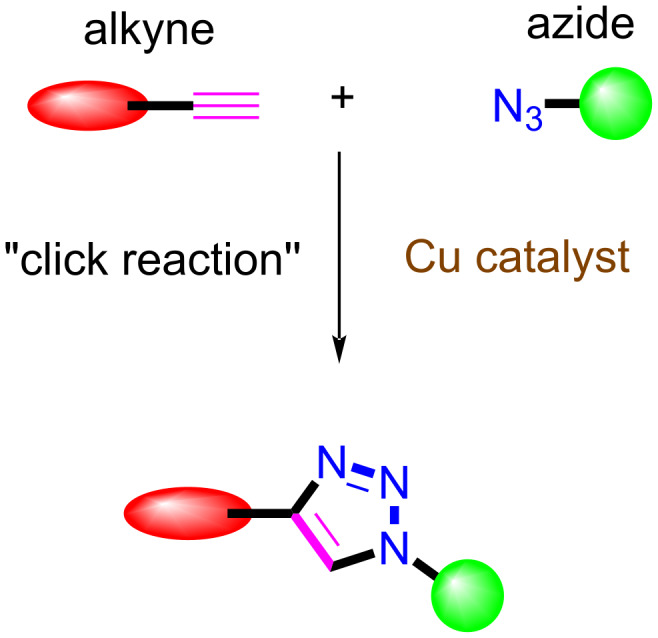
Alkyne–azide "click reaction".

In the past, many synthetic procedures have been utilized to covalently connect a porphyrin with a chromophoric group. Among these, the copper(I)-catalyzed Huisgen 1,3-dipolar cycloaddition reaction [1–2] of azides with terminal alkynes is a popular and well established process to link a porphyrin with other moieties via 1,2,3-triazole group [3] (Figure 1).

The term “click chemistry” was introduced for the first time by K. B. Sharpless in 1999 at the 217th ACS annual meeting [4]. Most recently, K. B. Sharpless shared the 2022 Nobel Prize in Chemistry with C. R. Bertozzi and M. Meldal for the development of click chemistry and bioorthogonal chemistry. Due to high product yields, quick reaction times, and excellent regioselectivity, the "click" cycloaddition reaction has enabled the synthesis of several compounds with a wide range of applications in a variety of sectors, including bioconjugation [5–6] drug development [7–8], glycoscience [9–10], porphyrin chemistry [11], nanoscience [12], and materials research [13–14].

**Figure 2:**
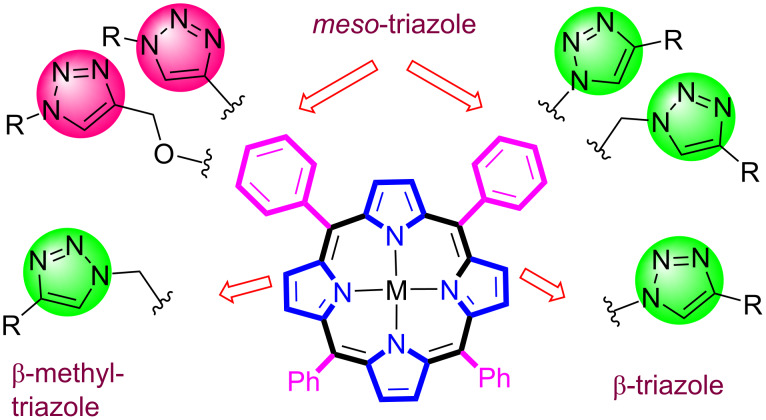
β- and *meso*-triazole-linked porphyrin.

Moreover, this review describes the use of the click methodology for the construction of various β- and *meso*-substituted 1,2,3-triazoloporphyrins by using azide or alkyne-substituted porphyrin as substrate (Figure 2). Every example mentioned in this review includes a brief synthetic procedure with reaction conditions, product yields, and photophysical and other properties of the end products.

## Review

### Overview of CuAAC click reactions on porphyrins

The CuAAC-inspired click reaction is particularly useful for the coupling of two different moieties comprising azide and alkyne groups in high yield and regioselectivity without using harsh reaction conditions. In recent years, this click protocol has been successfully employed in porphyrin chemistry to synthesize various triazoloporphyrins by attaching an azide or alkyne moiety to the periphery of the porphyrin. Since all of the triazole-linked porphyrins discussed in this review were prepared for different applications, most of the discussion focuses on the clicked synthetic parts under different reaction conditions that include different Cu catalysts, solvent systems, ligands, and temperatures. Most of the porphyrins used in the Cu(I)-catalyzed click reaction have been metalated prior to the reaction; otherwise, Cu metal can be inserted into the porphyrin cavity. As a result, the catalytic activity of Cu will decrease, it will be more difficult to remove, and more additional steps will be required to synthesize other corresponding metal derivatives. To this end, the review is divided into two major sections, the first of which describes the synthesis of β-triazole-bridged porphyrin conjugates and the second of which discusses the preparation of *meso*-triazole-linked porphyrin conjugates. Because the literature on *meso*-substituted triazole-bridging porphyrin synthesis is more than that of β-substituted triazoloporphyrin, therefore, the second section is further divided into five subsections to discuss the synthesis of *meso*-substituted mono-, di-, tri-, tetra-, and octatriazole-linked porphyrin conjugates.

### Synthesis of β-triazole-bridged porphyrin conjugates

**Scheme 1:**
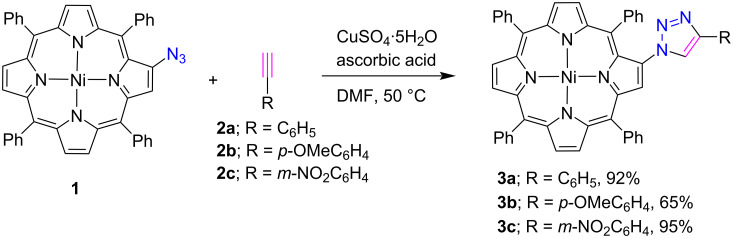
Synthesis of β-triazole-linked porphyrins **3a**–**c**.

In recent years, many β-triazole-linked porphyrin conjugates have been synthesized using the “click chemistry” protocol. For the synthesis of these compounds, an azide moiety was introduced at the β*-*position of various porphyrins. In 2007, Shen et al. [15] exploited the concept of “click chemistry” for the construction of β-substituted-triazoloporphyrins **3a–c** in 65–95% yield by the reaction of β-azidotetraphenylporphyrins **1** with various arylalkynes **2a–c** via copper(I)-catalyzed Huisgen 1,3-dipolar cycloaddition reaction in DMF at 50 °C in the presence of CuSO_4_·5H_2_O and ascorbic acid as shown in Scheme 1. It was noted that an alkyne with an electron-withdrawing group **2c** was found to be more reactive than an electron-donating group **2b** in the formation of triazoloporphyrins.

**Scheme 2:**
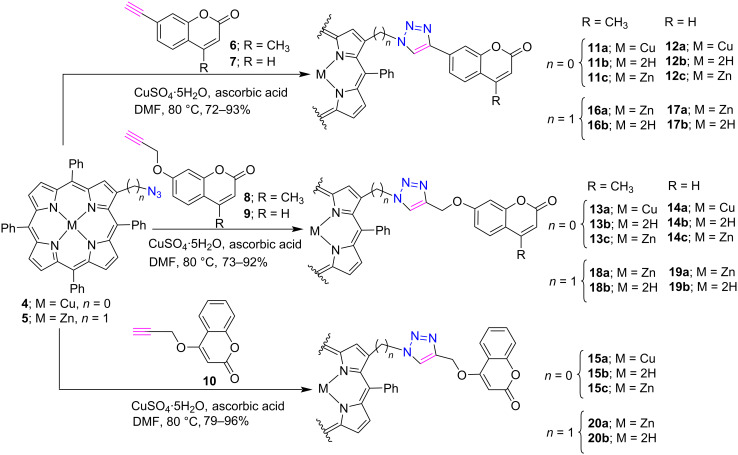
Synthesis of β-triazole-bridged porphyrin-coumarin conjugates **11–20**.

Similar to porphyrins, coumarin and its derivatives also possess diverse biological as well as optical properties. Moreover, the versatility of coumarin hybrids finds numerous applications including fluorescent brightening agents [16], optical sensors [17], organic light emitting diodes [18–19], light harvesting materials [20] and fluorescent probes in biological imaging [21]. Therefore, keeping the biological and optical properties of coumarin and porphyrin in mind, in 2015 we reported the synthesis and photophysical studies of β-triazole-bridged porphyrin-coumarin conjugates **11–15** [22] and β-triazolomethyl-linked porphyrin-coumarin dyads **16–20** [23] by using a click reaction approach. First, copper and zinc derivatives of the porphyrin-coumarin conjugates **11a–20a** were synthesized in excellent yields by the click reaction between copper(II)-2-azido-5,10,15,20-tetraphenylporphyrin (**4**) or zinc(II)-2-azidomethyl-5,10,15,20-tetraphenylporphyrin (**5**) and various alkyne-substituted coumarins **6–10** in the presence of CuSO_4_·5H_2_O and ascorbic acid in DMF at 80 °C (Scheme 2). Further, the corresponding free-base porphyrins **11b–20b** were obtained in good yields after demetallation of copper and zinc porphyrins under acidic conditions. Also, their zinc analogues **11c–15c** were obtained by the treatment of free-base porphyrins with zinc acetate. The photophysical studies of these synthesized conjugates revealed that some of them show substantial intramolecular energy transfer between porphyrin and coumarin moieties.

**Scheme 3:**
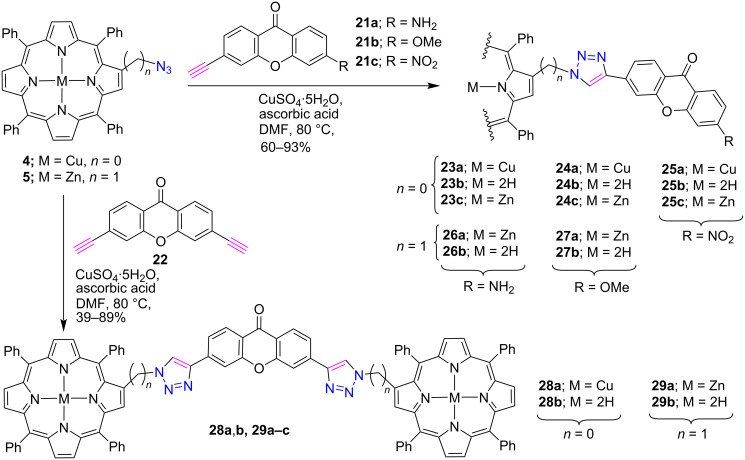
Synthesis of β-triazole-bridged porphyrin-xanthone conjugates **23–27** and xanthone-bridged β*-*triazoloporphyrin cojugates **28** and **29**.

Similar to coumarins, synthetic and naturally occurring xanthones also possess many biological [24] as well as fluorescent properties [25]. Therefore, considering these properties of xanthone, we used a click chemistry protocol to prepare β*-*triazole-linked porphyrin-xanthone conjugates **23–27** and xanthone-bridged β*-*triazolobisporphyrin conjugates **28**, **29** [26] (Scheme 3). Initially, the click reaction was performed between copper azidoporphyrin **4** or zinc porphyrin **5** with various alkyne-substituted xanthones **21a–c** and **22** for the construction of porphyrins **23a–29a**. Furthermore, these metalated porphyrins underwent demetallation under acidic conditions to give the corresponding free-base porphyrins **23b–29b** in good yields. In addition, their zinc analogues **23c–25c** were also obtained in excellent yields by the reaction of free-base porphyrins with zinc acetate. The preliminary photophysical studies of the porphyrin-xanthone dyads show a bathochromic shift in the absorption and fluorescence spectra.

### Synthesis of *meso-*triazole-bridged porphyrin conjugates

In recent years, many chromophoric moieties have been introduced at the *meso*-position of porphyrins by implying diverse synthetic procedures. Among these, the CuAAC click-mediated reactions are proving to be a popular choice for the construction of *meso*-substituted triazoloporphyrins. For this purpose, an azide or alkyne group was incorporated either on the *meso*-phenyl ring or on an alkyl chain of various porphyrin derivatives. This section covers the synthesis of porphyrin conjugates containing one, two, three, four, and eight 1,2,3-triazole units.

#### Mono-triazole-bridged porphyrin systems

**Scheme 4:**
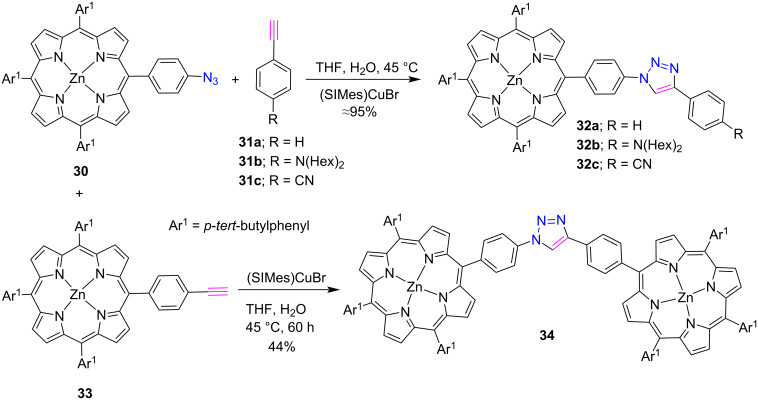
Synthesis of *meso*-triazoloporphyrins **32a–c** and triazole-bridged diporphyrins **34**.

This section will describe the synthesis of *meso*-triazole-linked porphyrins containing one triazole ring by using *meso*-substituted azide or alkyne bearing porphyrins. In 2007, Séverac et al. [27] reported the preparation of *meso*-phenyl-linked triazoloporphyrins **32a–c** and triazole-bridged bisporphyrins **34** in good yields. The “click reaction” of azidoporphyrin **30** with the terminal alkynes **31a–c** and **33** in a THF/water (3:1) mixture was investigated by using different catalytic systems. Among these, copper carbene (SIMes)CuBr proved to be a better catalyst than other copper catalysts, as it provided higher yields of the desired products (Scheme 4).

**Scheme 5:**
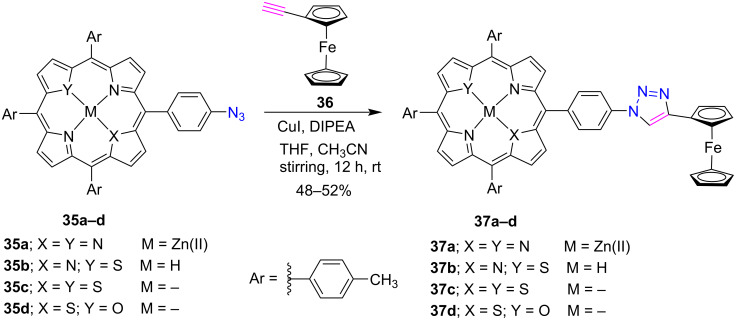
Synthesis of *meso*-triazole-linked porphyrin-ferrocene conjugates **37a–d**.

In 2010, Shetti and Ravikanth [28] nicely utilized the "click reaction" approach for the preparation of a series of triazole-bridged porphyrin-ferrocene dyads **37a–d** in 48–52% yield by the reaction between two clickable subunits, azido-porphyrins **35a–d** and alkyne-functionalized ferrocene **36** in the presence of CuI and DIPEA in THF/CH_3_CN (Scheme 5).

Interestingly, among the various copper(I)-catalyzed click reaction conditions, the best yields were obtained when CuI/DIPEA (0.1:1) was used in a THF/CH_3_CN mixture. In a photophysical investigation, a weak interaction was observed between two subunits at ground state in porphyrin-ferrocene dyads. Also, the fluorescence of porphyrin was significantly quenched in porphyrin-ferrocene conjugates due to photoinduced electron transfer from ferrocene to porphyrin subunits.

**Scheme 6:**
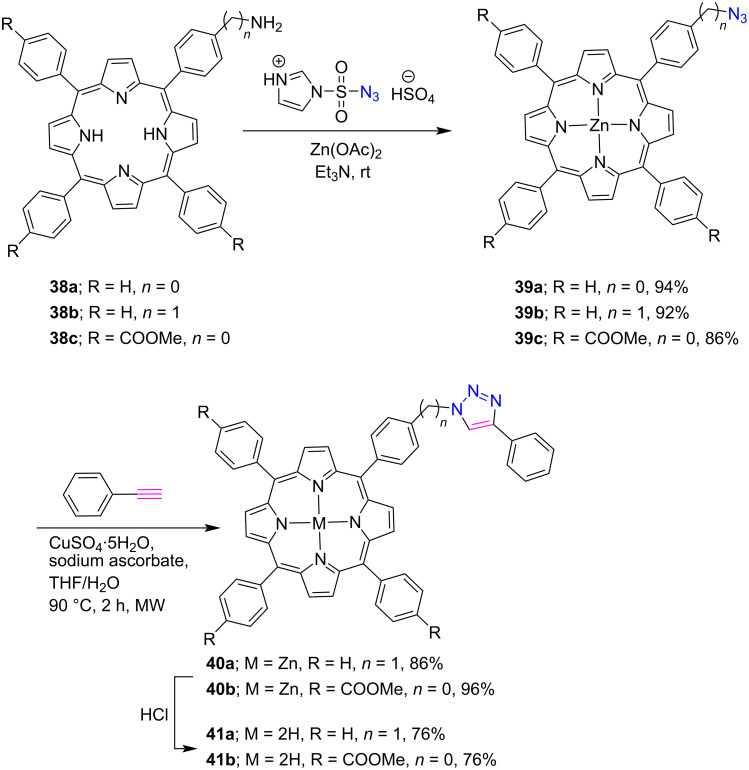
Synthesis of *meso*-triazole-linked porphyrin conjugates **40a**,**b** and **41a**,**b**.

Bryden and Boyle [29] presented a mild and facile one-pot synthesis of *meso*-substituted zinc(II) azido-porphyrin **39a–c** in excellent yield by using a diazo-transfer reagent and utilized it for the preparation of 1,2,3-triazole-linked porphyrin conjugates **40a**,**b** using copper(I)-catalyzed click reactions (Scheme 6). The authors selected the hydrogen sulfate salt of imidazole-1-sulfonyl-azide for the formation of azide porphyrins **39a–c** due to its greater shock stability and storage than its HCl salt. After the successful preparation of the zinc triazoloporphyrins **40a**,**b**, their corresponding free-base analogues **41a**,**b** were also obtained by the treatment with HCl in dioxane.

**Scheme 7:**
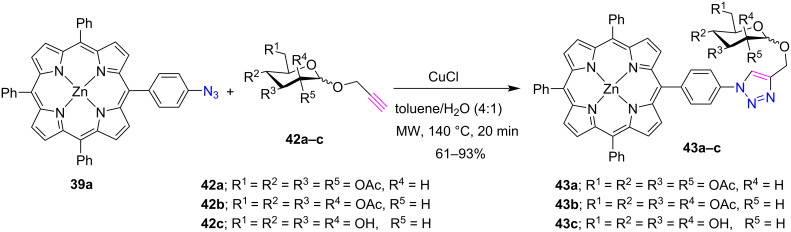
Synthesis of *meso*-triazole-linked glycoporphyrins **43a–c**.

The conjugation of carbohydrate with porphyrin improves the solubility of porphyrin in aqueous medium and also improves the optical and medicinal properties of porphyrins [30]. Considering these properties of glycoporphyrins, Locos et al. [31] successfully used the Cu(I)-catalyzed click reaction to synthesize triazole-linked glycoporphyrins under microwave conditions. At first, the glycoporphyrins **43a**,**b** were synthesized in good yields by the reaction between zinc(II) 5-(4-azidophenyl)-10,15,20-triphenylporphyrin (**39a**) and protected α-propargylglucose **42a** or mannose **42b** in the presence of CuCl in a toluene/H_2_O (4:1) (Scheme 7). Further a fully deprotected mannosyl residue **42c** was also introduced via "click reaction”, yielding the glycoporphyrin **43c**. Moreover, this methodology can also be utilized to introduce various carbohydrate sequences suitable for tumor-binding-associated lectins on the porphyrin periphery.

**Scheme 8:**
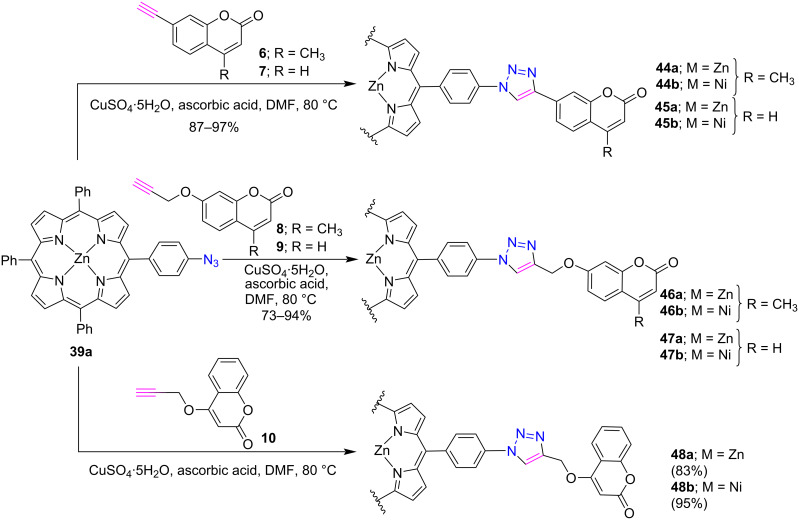
Synthesis of *meso*-triazole-linked porphyrin-coumarin conjugates **44–48**.

Continuing our work on the functionalization of porphyrin at the *meso*-position [32–33], we used the CuAAC click protocol to synthesize *meso*-phenyltriazole-linked porphyrin-coumarin dyads **44–48** in good to excellent yields. Firstly, Zn(II) *meso*-phenyltriazole-linked porphyrin-coumarin conjugates **44a–48a** were synthesized by using the “click reaction” between zinc(II) 5-(4-azidophenyl)-10,15,20-triphenylporphyrin (**39a**) and diverse alkyne-substituted coumarins **6–10** in DMF containing CuSO_4_·5H_2_O and ascorbic acid at 80 °C [34] (Scheme 8). Further, nickel derivatives **44b–48b** were also obtained from their zinc analogues by demetallation with concentrated HCl and metalation with nickel acetate. The preliminary photophysical results revealed a significant intramolecular energy transfer between the porphyrin core and the coumarin moiety in the case of zinc porphyrin analogues.

**Scheme 9:**
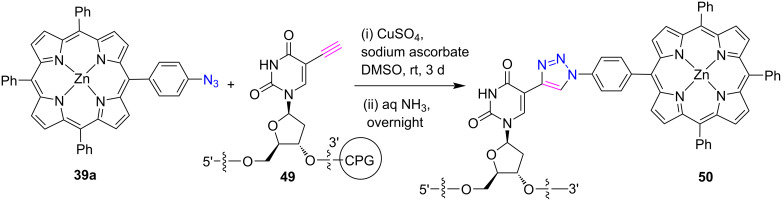
Synthesis of *meso*-triazole-bridged porphyrin-DNA conjugate **50**.

In 2017, Yamana et at. [35] reported the synthesis of porphyrin-DNA conjugate **50** by a solid-phase “click reaction” between azidoporphyrin **39a** and oligodeoxynucleotides **49** bearing an ethynyl group in the presence of CuSO_4_·5H_2_O and sodium ascorbate in DMSO (Scheme 9).

According to UV–vis and circular dichroism (CD) spectrum measurements, the authors state that the formation of the dimer of porphyrin **50** can be attributed to DNA hybridization and through-space electronic interactions. In this investigation, it was found that the DNA serves as a useful skeleton to regulate the relative location of the porphyrin molecules in order to produce the improved photocurrent response from the DNA modified electrodes.

**Scheme 10:**
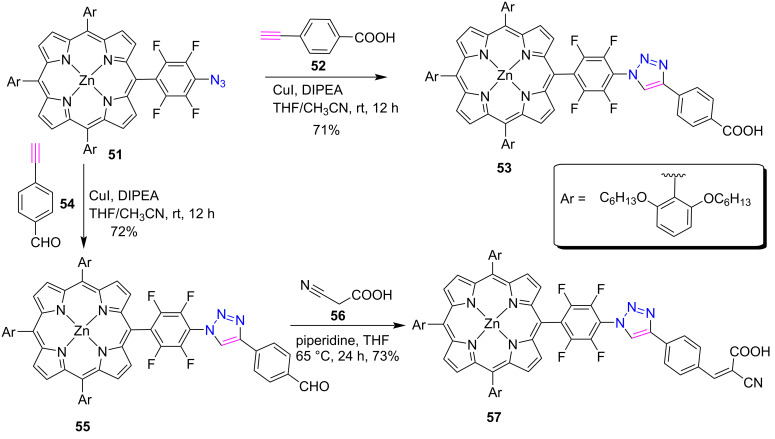
Synthesis of *meso*-linked porphyrin-triazole conjugates **53** and **57**.

In 2018, Coutsolelos et al. [36] established a “click chemistry” protocol to prepare *meso*-triazole-fused porphyrin conjugates **53** and **55** as sensitizers for dye-sensitized solar cells (DSSCs). These porphyrin conjugates were synthesized through the click reaction between azidoporphyrin **51** and alkynes **52** or **54**. Further, porphyrin **57** bearing a CN and a COOH group was prepared by the treatment of porphyrin **55** with 2-cyanoacetic acid (**56**) in piperidine as shown in Scheme 10.

To prepare these conjugates, a porphyrin with a pentafluorophenyl ring (because of its strong electron-withdrawing ability) was chosen and used in the synthesis of azidoporphyrin **51** by replacing the *para*-fluoro atom with an azide group. Interestingly, the photovoltaic device performance of porphyrins **53** and **57** was increased by eight-fold and four-fold, respectively, as compared to reference compounds without a triazole ring.

**Scheme 11:**
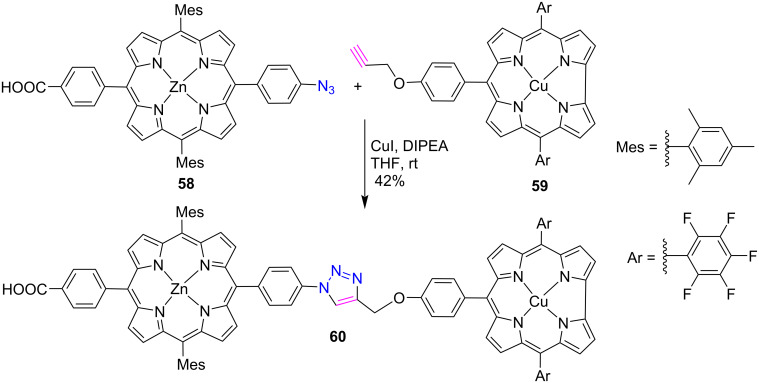
Synthesis of *meso*-triazole-linked porphyrin-corrole conjugate **60**.

Likewise, Nikolaou et al. [37] used zinc porphyrin **58** and copper-corrole **59** to synthesize first porphyrin-corrole dyad **60** through click protocol in a 42% yield by using CuI and DIPEA in THF at room temperature (Scheme 11). Their photophysical studies revealed that the fluorescence of zinc porphyrin **58** is strongly quenched due to the copper-corrole **59** in the dyad **60**. Also, an efficient excited-state interaction was found between zinc-porphyrin and copper-corrole moieties upon photoexcitation. Moreover, the theoretical calculations suggested that the porphyrin moiety acted as a donor while the corrole unit acted as an acceptor in dimer **60**.

**Scheme 12:**
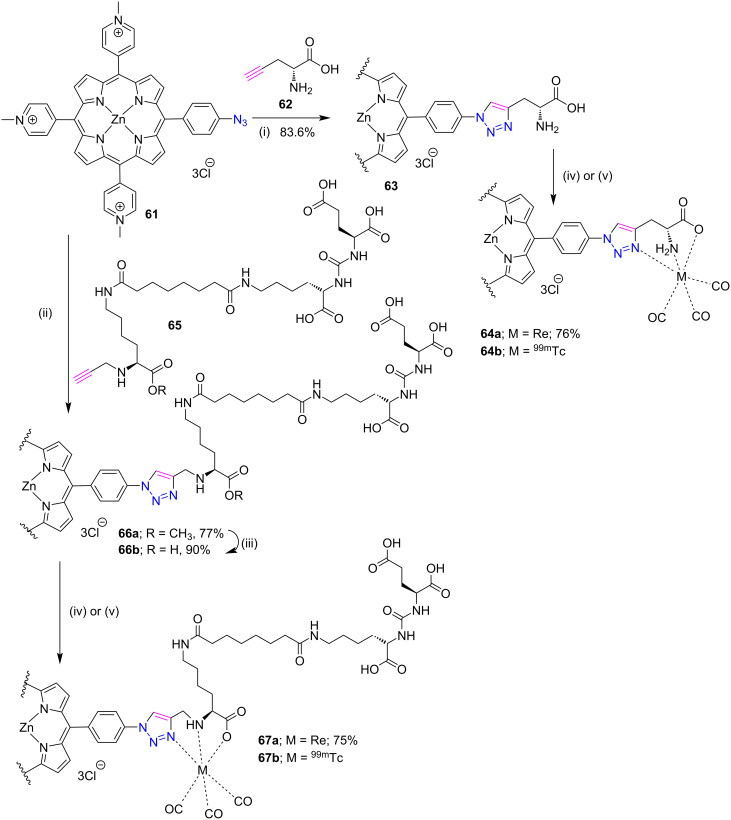
Synthesis of porphyrin conjugates **64a**,**b** and **67a**,**b**. Reaction conditions: (i) CuSO_4_, sodium ascorbate, MW, 50 °C (ii) CuSO_4_, sodium ascorbate, tris[(1-benzyl-1*H*-1,2,3-triazol-4-yl)methyl]amine, MW, 70 °C (iii) LiOH, water, room temperature, 3 h (iv) phosphate buffer pH 7, [Re(CO)_3_Br_3_][NEt_4_]_2_, 65 °C, 2 h (v) [^99m^Tc(CO)_3_(H_2_O)_3_]^+^, pH 7.4, 90 °C, 30 min.

Recently S. Y. Yap and co-workers [38] used “click chemistry” to link a water-soluble porphyrin with a histidine like group for the development of theranostic agents. Firstly, zinc triazole-porphyrins **63** and **66a** were synthesized by the click reaction between water-soluble azidoporphyrin **61** and ᴅ-propargylglycine **62** or trifunctional-propargyllysine **65** in 83.6% and 77% yield, respectively, under microwave conditions as described in Scheme 12. After the successful synthesis of porphyrins **63** and **66b**, their corresponding rhenium complexes **64a** and **67a** were prepared in good yields by using [Re(CO)_3_Br_3_][NEt_4_]_2_ at 65 °C in 2 hours. In addition to these, the radiolabeled products **64b** and **67b** were also constructed from the corresponding rhenium complexes by using [^99m^Tc(CO)_3_(H_2_O)_3_]^+^ at pH 7.4 and 90 °C temperature.

**Scheme 13:**
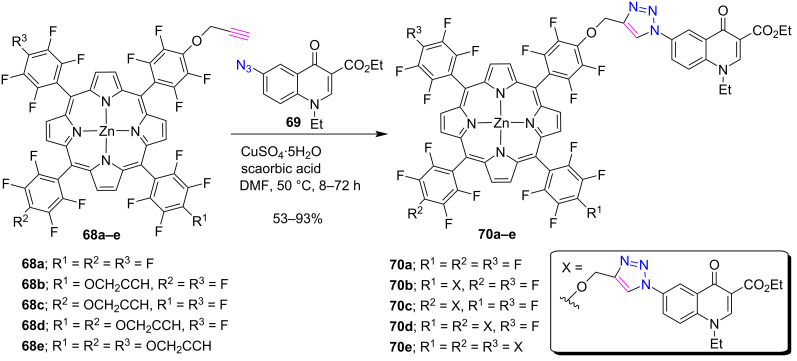
Synthesis of *meso-*triazole-bridged porphyrin-quinolone conjugates **70a–e**.

Santos et al. [39] reported in 2008 the synthesis of CuAAC-ensembled 1,2,3-triazole-linked porphyrin-quinolone conjugates **70a–e** by considering the biological significance of both the porphyrin and quinolone groups. The click reaction was performed between alkyne-substituted porphyrins **68a–e** and 6-azidoquinolone **69** for the preparation of mono-, di-, tri-, and tetratriazoloporphyrin-quinolone conjugates **70a–e** in 53–93% yields (Scheme 13).

**Scheme 14:**
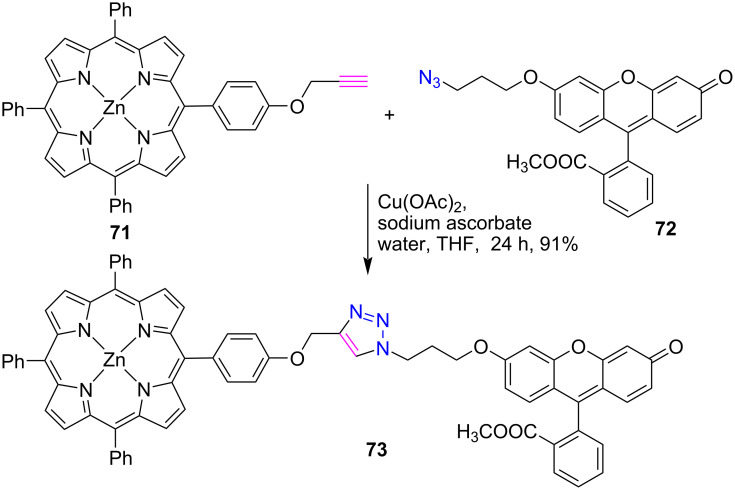
Synthesis of *meso*-triazole-linked porphyrin-fluorescein dyad **73**.

In the following reports, Leroy-Lhez et al. [40] used a similar synthetic protocol to link porphyrin **71** to a fluorescein **72** through a 1,2,3-triazole linker to form the porphyrin-fluorescein conjugate **73** in 91% yield (Scheme 14).

Furthermore, the fluorescence study of conjugate **73** revealed an intramolecular energy transfer between fluorescein and porphyrin subunits and was more efficient in DMSO as compared to chloroform. Also, a clear evidence of a folded conformer was found by electrostatic and CH–π interactions, which was also, confirmed by density functional theory (DFT) calculations.

**Scheme 15:**
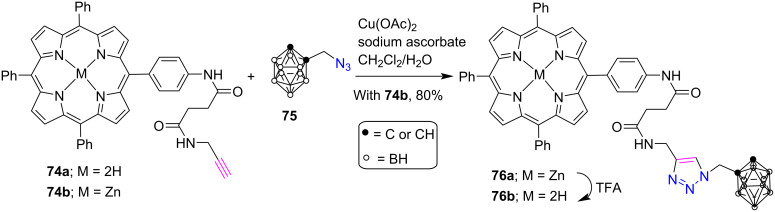
Synthesis of *meso*-triazole-linked porphyrin-carborane conjugates **76a**,**b**.

In another report, *meso*-triazole-bridged porphyrin-carborane conjugates **76a**,**b** were synthesized in 80% yield by Ol’shevskaya et al. where a porphyrin unit **74a** is covalently linked to 1-azidomethyl-*o*-carborane **75** [41] (Scheme 15). This boronation of the amide was performed via “click chemistry” in СН_2_Сl_2_/Н_2_О solvent system using Cu(OAc)_2_·2H_2_O and sodium ascorbate as the catalyst at room temperature. In addition, the free-base porphyrin-carborane conjugate **76b** was successfully obtained after the treatment of **76a** with trifluoroacetic acid in СН_2_Сl_2_.

**Scheme 16:**
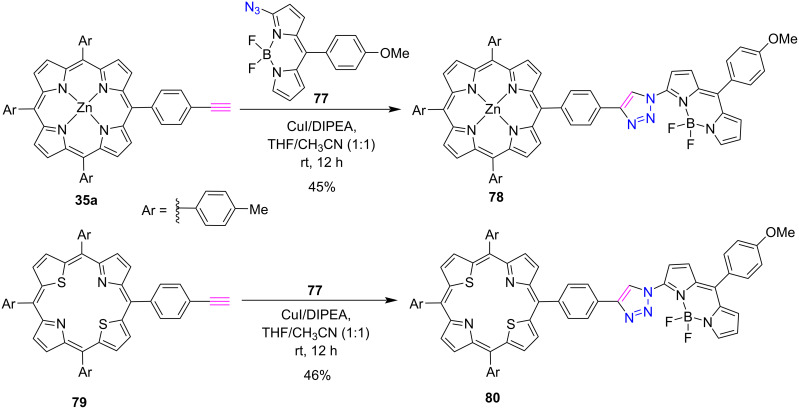
Synthesis of *meso***-**triazole-bridged porphyrin-BODIPY conjugates **78** and **80**.

The research group of Ravikanth [42] prepared the triazole-bridged porphyrin-BODIPY conjugates **78** and **80** via CuAAC reaction in 45–50% yields. This click reaction was performed between 3-azido-BODIPY **77** and zinc porphyrin **35a** or 21,23-dithiaporphyrin **79** in the presence of CuI/DIPEA in THF/CH_3_CN (Scheme 16). UV–vis absorption and electrochemical studies revealed the presence and interaction of both the moieties in the conjugates. In addition to this, the fluorescence studies suggested that the energy transfer was not efficient in Zn(II) porphyrin-BODIPY and 21,23-dithiaporphyrin-BODIPY conjugates.

**Scheme 17:**
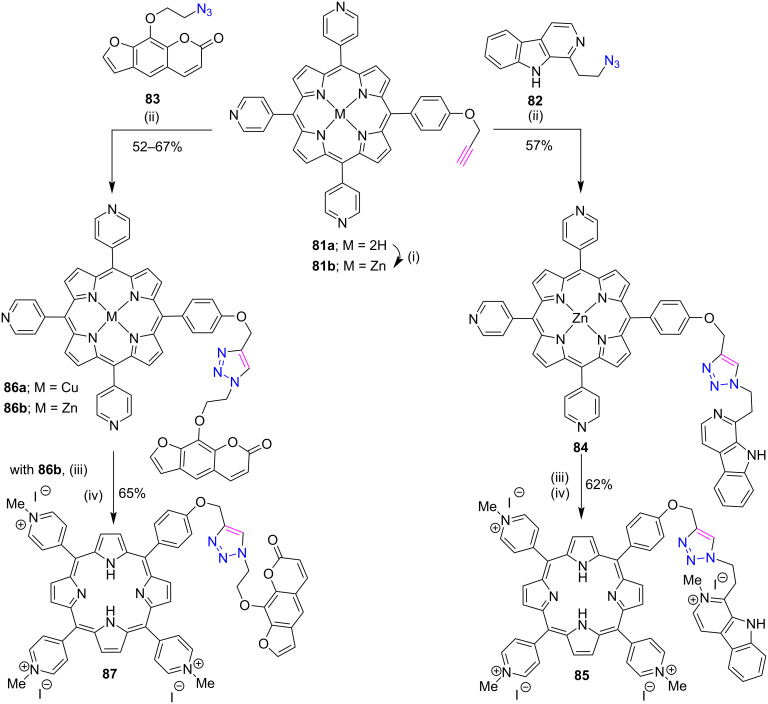
Synthesis of *meso***-**triazole-linked cationic porphyrin conjugates **85** and **87**. Reaction conditions: (i) Zn(OAc)_2_, CHCl_3_, MeOH, reflux, 2 h (ii) CuSO_4_∙5H_2_O, sodium ascorbate, DMF/H_2_O (1:1), 80 °C, 32–168 h (iii) CHCl_3_, aq HCl (25%), 1 h (iv) MeI (120 equiv), DMF, rt, 36–72 h.

Dalip Kumar and his co-workers reported a CuAAC click reaction to prepare novel water soluble triazole-linked cationic porphyrin-carboline conjugate **85** [43] and porphyrin-psoralen conjugates **87** [44] as shown in Scheme 17. Firstly, zinc(II) porphyrin-carboline conjugate **84** and porphyrin-psoralen conjugate **86b** were synthesized in good yields by the CuAAC reaction between porphyrin **81b** and azide **82** or **83**. Interestingly, the click reaction between free-base porphyrin **81a** and azide **83** gives Cu(II)-porphyrin-psoralen conjugates **86a** which do not give the demetallated product upon reaction with TFA. To this end, zinc porphyrins **84** and **86b** were treated with HCl to remove the metal from its cavity and were then methylated with MeI in DMF to afford the cationic products **85** and **87** in good yields. The synthesized conjugates exhibited high photocytotoxicity towards A549 cancer cells as compared to a tumor-localizing and potent photosensitizing agent, TMPyP. Further, the authors discovered that the porphyrin-carboline conjugates **85** and porphyrin-psoralen conjugate **87** could be potential candidates for PDT applications.

**Scheme 18:**
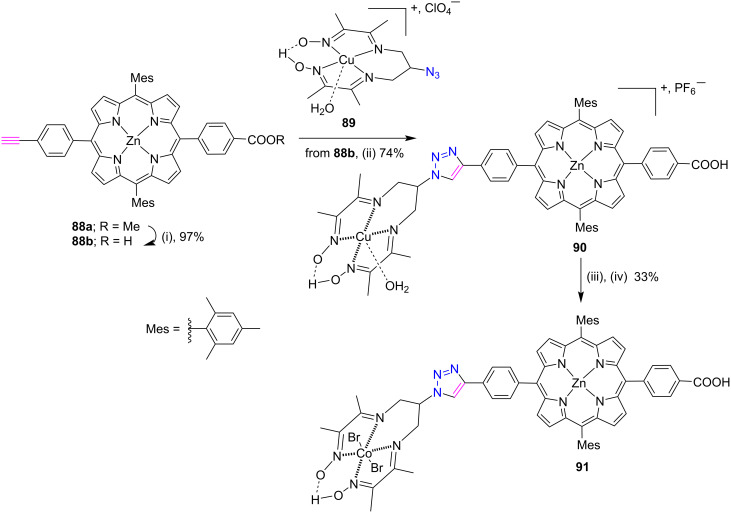
Synthesis of *meso*-triazole-cobalt-porphyrin diimine-dioxime conjugate **91**. Reactions conditions: (i) KOH, THF/MeOH/H_2_O, (ii) CuSO_4_·5H_2_O, sodium ascorbate, CH_2_Cl_2_/H_2_O/MeOH, rt, 24 h, aq PF_6_^−^, (iii) CoCl_2_∙6H_2_O, THF/acetone, rt, air bubbling, overnight, (iv) KBr, THF/acetone, rt, 5 d.

Recently, Charisiadis et al. [45] explored this modular click chemistry protocol for the synthesis of a noble metal-free *meso*-triazole cobalt porphyrin diimine-dioxime conjugate **91** by covalently connecting a zinc porphyrin sensitizer with a H_2_-evolving cobalt diimine dioxime catalyst. First, the Zn porphyrin **88b** was coupled with the copper diiminedioxime complex **89** via the CuAAC click reaction to give porphyrin complex **90** in 74% yield (Scheme 18). Furthermore, a stable Co(III) complex was obtained by the metal exchange reaction of porphyrin **90** with excess CoCl_2_·6H_2_O in an acetone/THF (1:1) mixture. The results of the photophysical investigations demonstrated that these dyads exhibit adequate electronic interactions between the sensitizer and catalyst in the excited state. In this study, NiO films were sensitized with porphyrin **91** and used in a photoelectrochemical cell for H_2_ generation. The NiO electrode is a transparent p-type conducting oxide and is used for hole injection from the HOMO of the excited dyes [46].

**Scheme 19:**
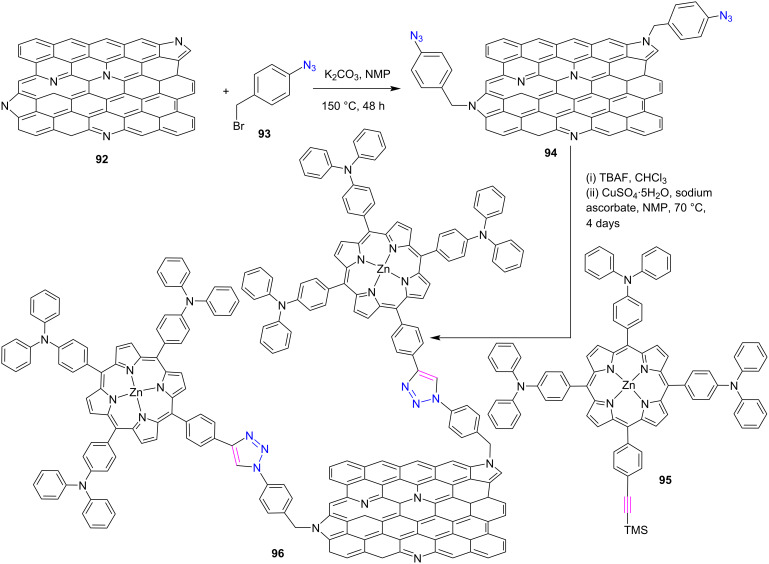
Synthesis of triazole-linked porphyrin-bearing N-doped graphene hybrid **96**.

Very recently, Arellano et al*.* [47] synthesized the *meso*-triazole-bridged zinc porphyrin-N-doped graphene hybrid **96** through the CuAAC reaction. For the click reaction synthesis of hybrid **96**, azide-functionalized N-doped graphene **94** and TMS-protected porphyrin **95** bearing an alkyne group were first prepared, as shown in Scheme 19. Subsequently, deprotected porphyrin bearing a terminal alkyne subunit was obtained after treatment of porphyrin **95** with TBAF. Finally, the CuAAC reaction was performed for the synthesis of triazole conjugates **96** by the reaction between acetylenic porphyrin with NG-N_3_ **94** in the presence of CuSO_4_·5H_2_O and sodium ascorbate in *N*-methyl-2-pyrrolidone (NMP). The fluorescence investigation of hybrid **96** revealed that the fluorescence of zinc porphyrin was strongly quenched, and the electron-transfer quenching mechanism was validated by easy oxidation. Furthermore, femtosecond transient-absorption spectrum investigations offered proof of the charge-separation process. The ultrafast pump-probe technique was used to determine the photoinduced charge separation in zinc porphyrin **96** and the rate constant was found on the order of 10^10^ s^−1^, clearly showing ultrafast electron-transfer between graphene and porphyrin units. Hence, these hybrids could be useful for light-induced applications.

**Scheme 20:**
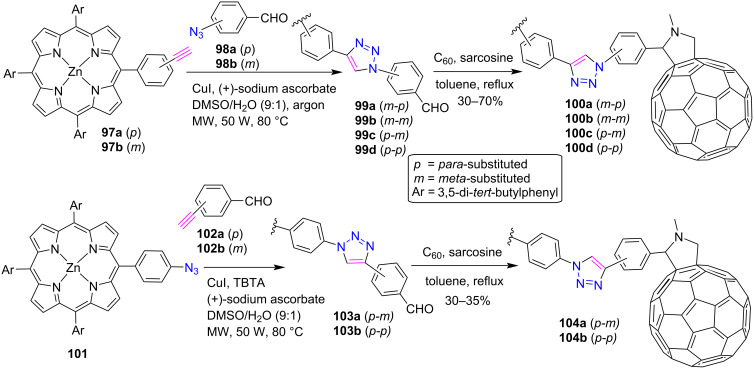
Synthesis of *meso*-triazole-linked porphyrin-fullerene dyads **100a–d** and **104a**,**b**.

Similar to porphyrins, fullerene C_60_ also possesses superior acceptor properties, low reduction potential and low reorganization energy [48–49]. Thus, keeping the diverse properties of fullerene in mind, de Miguel et al. [50] successfully prepared and characterized the triazole-linked porphyrin-fullerene dyads **100a–d** and **104a**,**b**. Firstly, *meso*-triazole-linked porphyrin conjugates **99a–d** and **103a**,**b** were synthesized from porphyrins **97a**,**b** and **101**, as shown in Scheme 20. For the click reaction between porphyrin **101** and alkyne **102a**,**b**, one equivalent of tris(benzotriazolylmethyl)amine (TBTA) was also added as an additive to increase the yield of triazoloporphyrins **103a**,**b**. When TBTA, a tertiary amine with a 1,2,3-triazole ring, is added to a Cu-catalyzed click reaction, it forms a complex to stabilize the Cu(I) oxidation state and speeds up the reaction. Further, these 1,2,3-triazole conjugates (**99a**–**d**, **103a**,**b**) were utilized for the preparation of porphyrin-fullerene dyads **100a–d** and **104a**,**b** in 30–70% yield by the reaction with C_60_ and sarcosine in toluene under microwave conditions.

The photophysical and computational studies of these ZnP-Tri-C_60_ conjugates revealed that the 1,2,3-triazole linker significantly influences the charge separation, charge recombination, and photoinduced electron transfer processes. Since, the through-bond distance is approximately the same for all the synthesized ZnP-Tri-C_60_ conjugates, therefore, the variation in charge separation and charge recombination dynamics is primarily related to the electronic properties of the conjugates, such as electron affinity, orbital energies, and excited state energies.

**Scheme 21:**
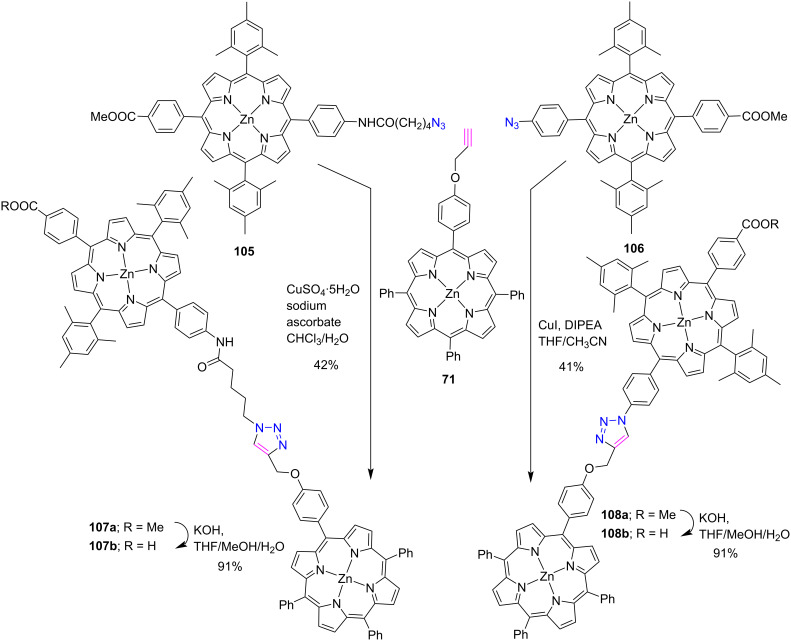
Synthesis of *meso*-triazole-bridged diporphyrin conjugates **107** and **108**.

In another report, Nikolaou et al. [51] described the synthesis of two novel porphyrin dyads **107b** and **108b** containing terminal carboxylic acid groups by using a CuAAC click reaction between azide and acetylene-substituted porphyrins in good yields as shown in Scheme 21. The photophysical and electrochemical studies along with DFT calculations showed that these dyads possess suitable frontier orbital energy levels for the use as sensitizers in DSSCs. Moreover, porphyrin conjugates **107b**- and **108b**-based solar cells were fabricated, resulting in power conversion efficiencies (PCEs) of 3.82 and 5.16%, respectively. In addition, the authors discovered that dyads **108b** containing shorter triazole bridges have a stronger absorption profile and higher dye loading of the corresponding solar cell.

**Scheme 22:**
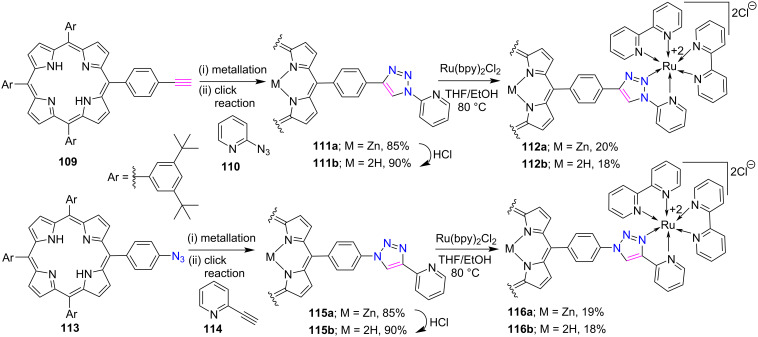
Synthesis of porphyrin-ruthenium (II) conjugates **112a**,**b** and **116a**,**b**. Reaction conditions: (i) Zn(OAc)_2_, CHCl_3_/MeOH (ii) CuSO_4_·5H_2_O, sodium ascorbate, DIPEA, CH_2_Cl_2_/EtOH/H_2_O, 50 °C.

Recently, Chauhan and co-workers [52] demonstrated a click-chemistry-inspired synthesis of porphyrin-*meso*-triazole-ruthenium(II) conjugates, as shown in Scheme 22. First, the porphyrin conjugates **111a**,**b** (inverse tri-py) and **115a**,**b** (regular tri-py) were prepared in 85–90% yield from their corresponding *meso-*substituted (*p*-alkynyl- or *p*-azidophenyl)porphyrins (**109** or **113**) under click reaction conditions. Further, these zinc and free-base *meso*-triazole-bridged porphyrin conjugates were reacted with *cis*-Ru(bpy)_2_Cl_2_ in THF to afford the porphyrin-*meso*-triazole-ruthenium(II) conjugates **112a**,**b** and **116a**,**b** in 18–20% yield. Their photophysical and electrochemical studies revealed that the orbital energies depend on the ligands/linker, connecting pattern of linkers, and the presence of Zn metal ions in the porphyrin core. Ligand exchange studies also suggested that the regular Ru(II)-tri-py complex are more stable than their inverse analogues.

#### Ditriazole-bridged porphyrin system

**Scheme 23:**
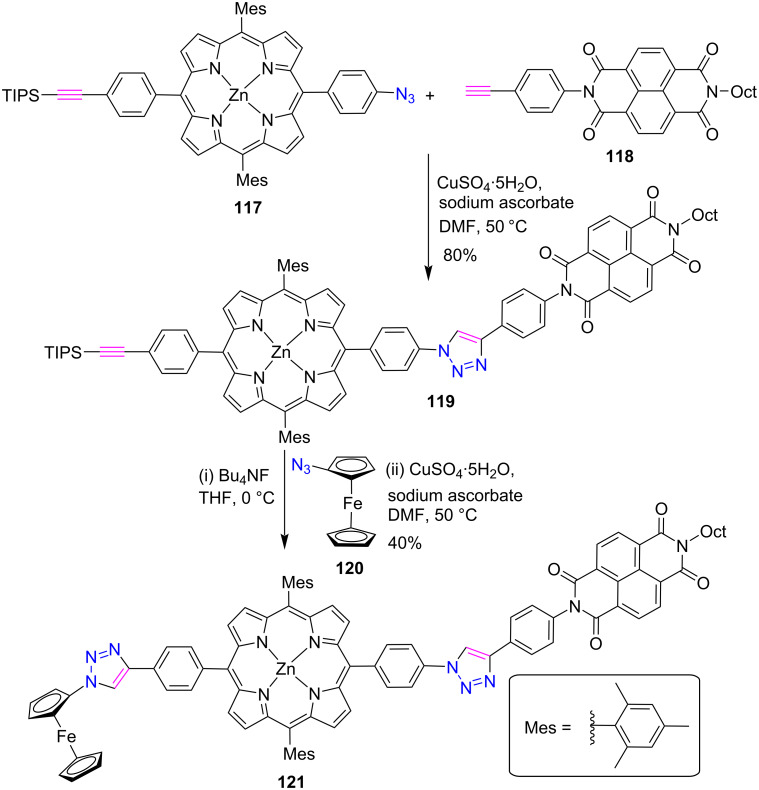
Synthesis of *meso*-triazole-linked porphyrin dyad **119** and triad **121**.

This section describes the synthesis of porphyrin conjugates having two triazole units. In a report, Natali et al. [53] synthesized the novel dyad **119** (ZnP-NDI) and triad **121** (Fc-ZnP-NDI) via “click reaction” containing a zinc porphyrin (ZnP), a naphthalenediimide (NDI) and a ferrocene (Fc) moiety (Scheme 23). Firstly, dyad **119** was synthesized in 80% yield by the reaction between azido-Zn-porphyrin **117** and naphthalenediimide **118** containing an alkyne moiety via CuAAC reaction and was subjected to deprotection of the triisopropylsilyl group with tetra-*n*-butylammonium fluoride (Bu_4_NF). Similarly triad **121** was prepared by the click reaction between dyads **119** and azido-ferrocene **120** in a 40% overall yield. The photophysical investigation revealed that dyad **119** exhibited relatively inefficient quenching of the ZnP singlet excited state, slow charge separation, and fast charge recombination processes on the visible excitation of ZnP and UV excitation of NDI chromophores. On the other hand, excitation of the NDI chromophore leads to the charge separation via both singlet and triplet quenching pathways.

**Scheme 24:**
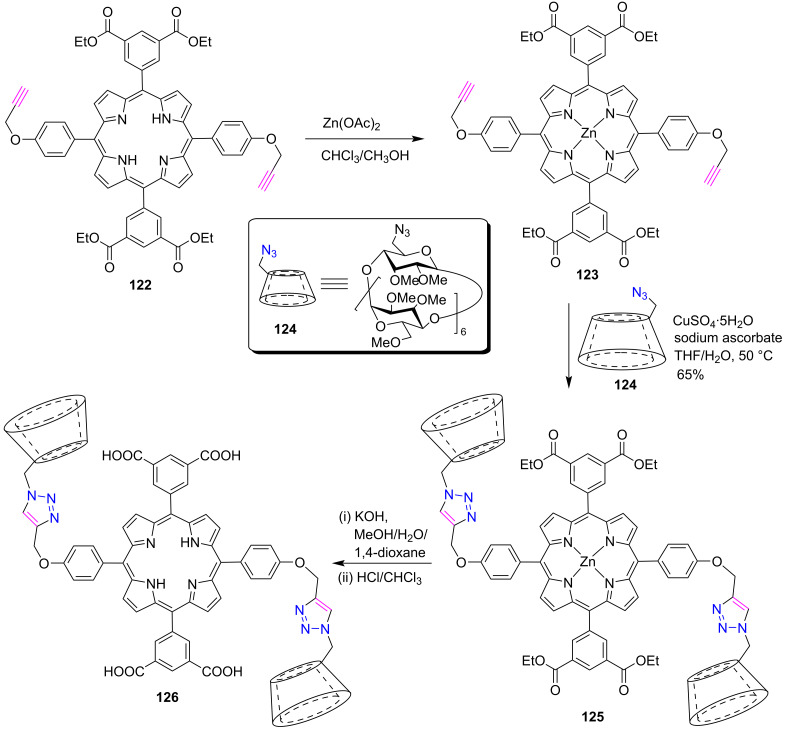
Synthesis of di-triazole-bridged porphyrin-β-CD conjugate **126**.

The construction of water-soluble porphyrin nanospheres was reported by Zhang et al. in 2014 [54] by intramolecular host–guest interactions using porphyrin **126** containing two permethyl-β-cyclodextrin (PM-β-CD) and two dicarboxylatophenyl arms at *meso*-positions. The synthesis of porphyrin **126** was accomplished in four steps as shown in Scheme 24. First, porphyrin **123** was obtained by zinc metalation of the free-base porphyrin **122** using zinc acetate in a chloroform-methanol mixture. Further, the azide–alkyne click reaction between porphyrin **123** and 6-azido-6-deoxy-PM-β-CD **124** in the presence of CuSO_4_ and sodium ascorbate in THF/H_2_O furnished the bis-triazole-bridged porphyrin-cyclodextrin conjugate **125** in 65% yield. Finally, porphyrin **126** was obtained from porphyrin **125** after ester hydrolysis with KOH and demetallation with concentrated HCl. After the successful synthesis of porphyrin **126**, it was used to form water-soluble porphyrin nanospheres by self-inclusion complex formation in water.

#### Tritriazole-bridged porphyrin system

**Scheme 25:**
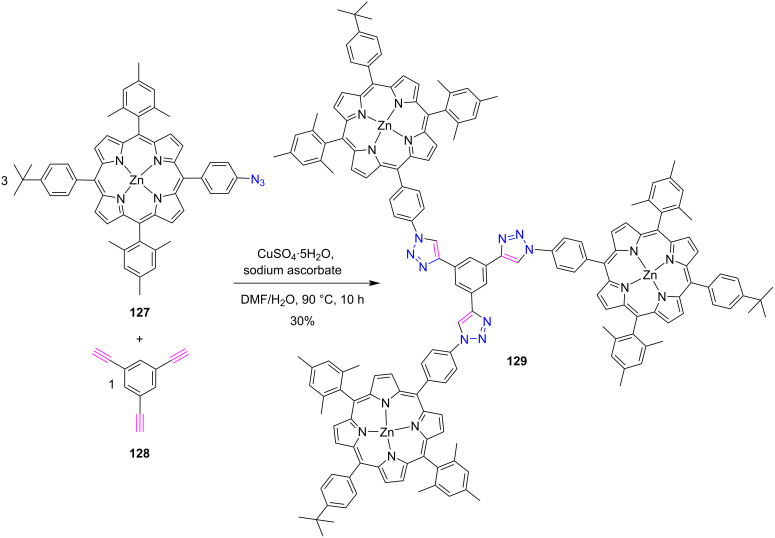
Synthesis of *meso*-triazole-bridged porphyrin star trimer **129**.

This section contains the synthesis of porphyrin conjugates having three 1,2,3-triazole groups as linkers. In 2012, Beletskaya and co-workers [55] described the synthesis of the novel 1,3,5-benzene-centered and triazole-bridged porphyrin star trimer **129** (Scheme 25) in 30% yield via CuAAC click reaction*.* In the UV–vis absorption spectrum, the absorption bands of product trimer **129** and starting compound **127** were almost coincided with each other, which suggested a weak intramolecular inter-porphyrin communication. Further investigation also revealed that the interaction of the star compound **129** with DABCO and Bipy possibly led to the double star formed by the axial coordination of ligand with zinc centers.

**Scheme 26:**
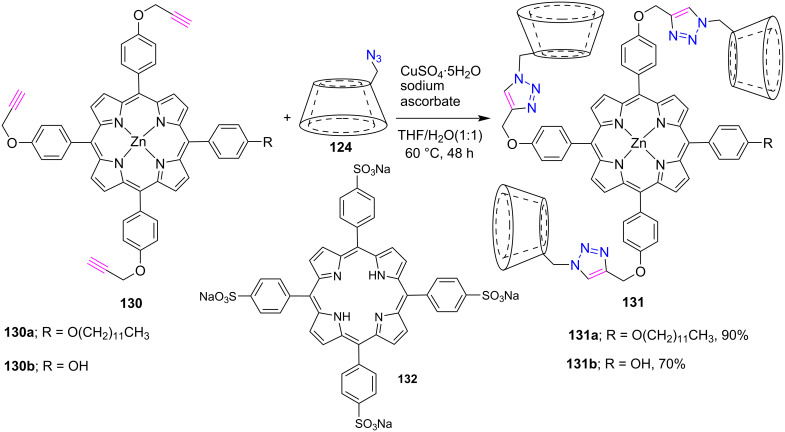
Synthesis of 1,2,3-triazole-linked porphyrin-β-CD conjugates **131a**,**b**.

The synthesis of supramolecular nanoassemblies from an amphiphilic 1,2,3-triazole-bridged porphyrin-permethyl-β-CD conjugates **131a**,**b** was reported by Liu et al. [56] in 2015. First the porphyrin-cyclodextrin conjugates **131a**,**b** were obtained in 90% and 70% yields, respectively, by the CuAAC click reaction between zinc(II) *meso*-tris(ethynylphenyl)porphyrin **130a**,**b** and 6-deoxy-6-azido-PM-β-CD **124** using copper sulfate and sodium ascorbate in THF/H_2_O (Scheme 26). Furthermore, the supramolecular nanoarchitectures as potential candidates for controlling drug delivery were prepared by complexing the porphyrins **131a**,**b** with tetrasodium tetraphenylporphyrintetrasulfonate **132**.

**Scheme 27:**
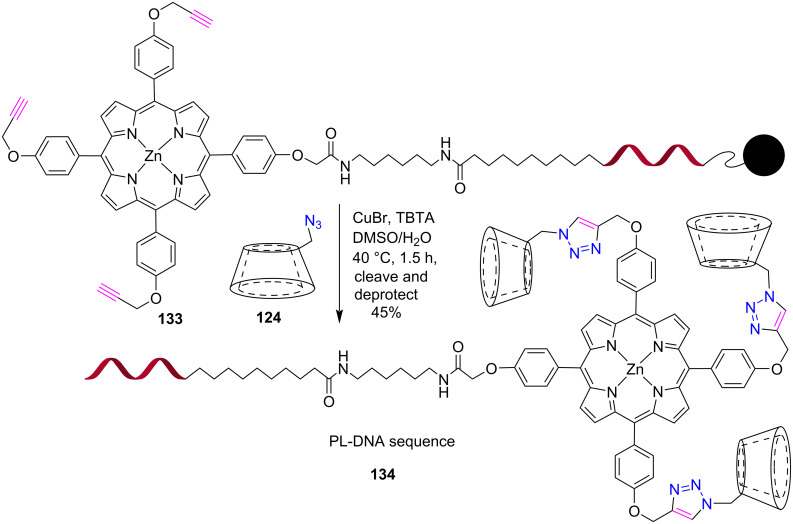
Synthesis of tritriazole-bridged porphyrin-lantern-DNA sequence **134**.

Recently, Pathak et al*.* [57] reported the synthesis of porphyrin-lantern-functionalized bright G-quadruplex nanostructures via a “tether and mask” approach in water (Scheme 27).

In this study, a large porphyrin dye **133** was first attached to short G-rich sequences and then clicked with azide **124** in the presence of copper bromide and tris((1-benzyl-4-triazolyl)methyl)amine (TBTA) in DMSO/H_2_O to give a porphyrin-lantern (PL)-DNA sequence in 45% yield after cleavage and deprotection. These PL-DNA sequences were further used to construct strong and fluorescent G-wires that could be useful for photonics applications.

#### Tetratriazole-bridged porphyrin system

**Scheme 28:**
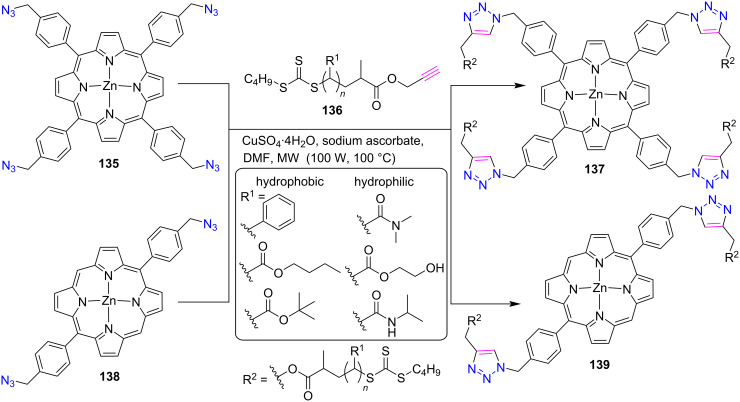
Synthesis of *meso***-**triazole-linked porphyrin-polymer conjugates **137** and **139**.

In this section, the click chemistry protocol for the synthesis of tetratriazole-bridged conjugates has been discussed. In the following report [58], Roberts et al. explored the click approach and designed the synthesis of hydrophobic and hydrophilic triazole-linked porphyrin-polymer conjugates (PPCs) (Scheme 28). The PPCs **137** and **139** were prepared by the reaction between tetraazido-Zn-porphyrin **135** or diazido-Zn-porphyrin **138** with polymers **136** bearing various hydrophilic and hydrophobic substituents using CuSO_4_∙4H_2_O and ascorbic acids under microwave irradiation conditions. The prepared nanoparticles from free-base PPCs showed colorimetric aqueous pH sensing, which suggests that the PPC nanoparticles could serve as chemical sensing applications.

**Scheme 29:**
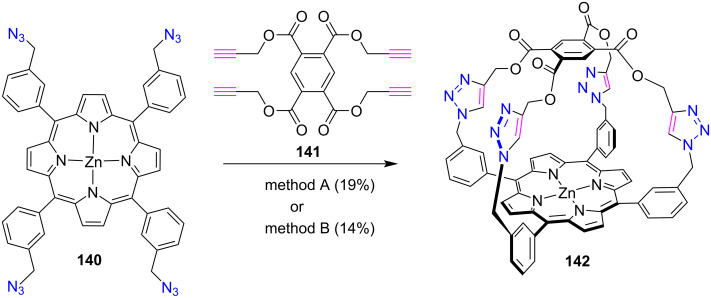
Synthesis of triazole-linked capped porphyrin **142**; Reaction conditions: method A: 10% H_2_O in THF, CuSO_4_·5H_2_O, ʟ-ascorbic acid, Et_3_N, reflux, 30 h; method B: DMF, Cu(MeCN)_4_PF_6_, Et_3_N, 70 °C, 16 h.

In 2008, the Crossley’s group [59] reported the synthesis of a ‘capped’ porphyrin **142** forming a rigid 1,2,3-triazole linker between the porphyrin and the capping group using the CuAAC click reaction (Scheme 29). Capped porphyrin **142** was synthesized in similar yields under both aqueous (method A) and anhydrous (method B) conditions.

Method A produced capped porphyrin **142** in 19% yield by clicking zinc(II) tetraazidoporphyrin **140** and tetraalkyne **141** in the presence of CuSO_4_∙5H_2_O, ʟ-ascorbic acid, and triethylamine in H_2_O/THF (1:10). In this procedure, the tertiary amine base was used to increase the rate of the CuAAC reaction. In contrast, method B reported the synthesis of porphyrin **142** with 14% yield using Cu(MeCN)_4_PF_6_ and triethylamine in anhydrous DMF. This ‘capped’ porphyrin was designed so that the rigid triazole linkers prevent the capping group from collapsing onto the porphyrin. Gilday et al. [60] also synthesized porphyrin **142** and studied its anion-binding properties and found that it has very strong anion-binding affinities for various anions.

**Scheme 30:**
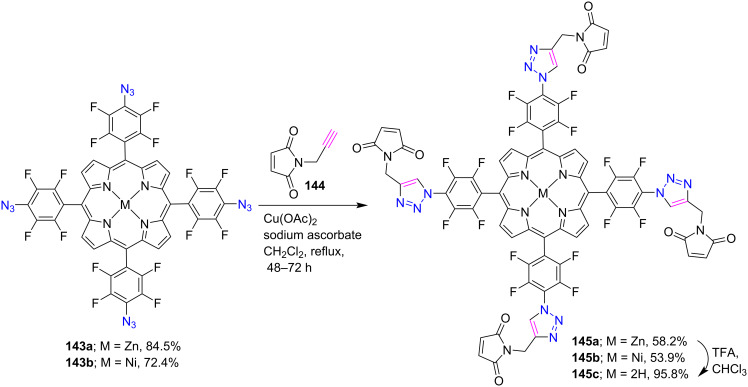
Synthesis of *meso*-tetratriazole-linked porphyrin-maleimine conjugates **145a**–**c**.

In 2019, Ol’shevskaya [61] and co-workers synthesized *meso*-tetratriazole-bridged fluorinated porphyrin-maleimine conjugates **145a–c** in 54–58% yields by using the CuAAC reaction between azidoporphyrins **143a**,**b** and *N*-propargylmaleimide (**144**) in CH_2_Cl_2_. In addition, the free-base counterpart **145c** was also prepared in quantifiable yield by removing zinc from porphyrin **145a** using CF_3_COOH in CHCl_3_ (Scheme 30).

**Scheme 31:**
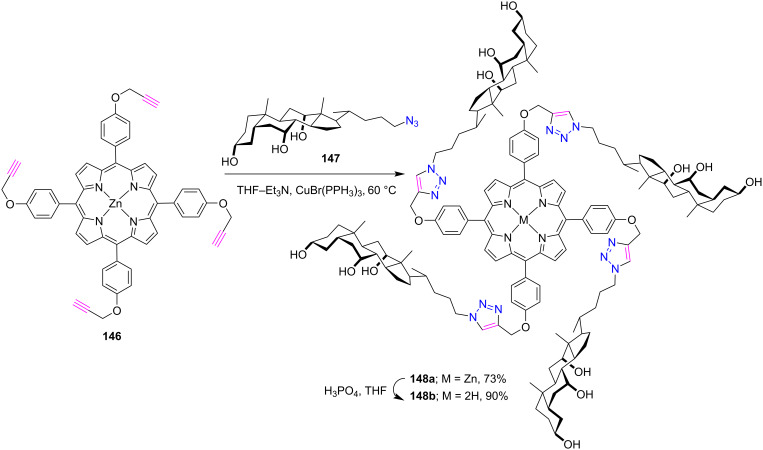
Synthesis of *meso*-tetratriazole-linked porphyrin-cholic acid complex **148a**,**b**.

In a report, Aguilar-Ortíz and co-workers [62] used the CuAAC tool for the synthesis of novel dendritic molecules **148a**,**b** containing a porphyrin and peripheral cholic acid moiety (Scheme 31). At first, the zinc derivative of porphyrin-tetracholic acid conjugate **148a** was synthesized by the CuAAC click reaction between porphyrin **146** and 3α,7α,12α-trihydroxy-24-azido-5β-cholane (**147**) in the presence of CuBr(PPh_3_)_3_ in Et_3_N/THF mixture in 73% yield.

Furthermore, this zinc conjugate was demetallated using H_3_PO_4_ in THF to form the free-base porphyrin **148b** in 90% yield. The photophysical properties of this dendronized porphyrin molecule was investigated by absorption and fluorescence spectroscopy in different solvents, and the amphiphilic properties of the cholic acid units in porphyrin conjugates were also studied. The zinc-metalated porphyrin-cholic acid conjugate tends to form J-aggregates in various solvents with different polarities, whereas demetallated porphyrin-cholic acid conjugates show different patterns.

**Scheme 32:**
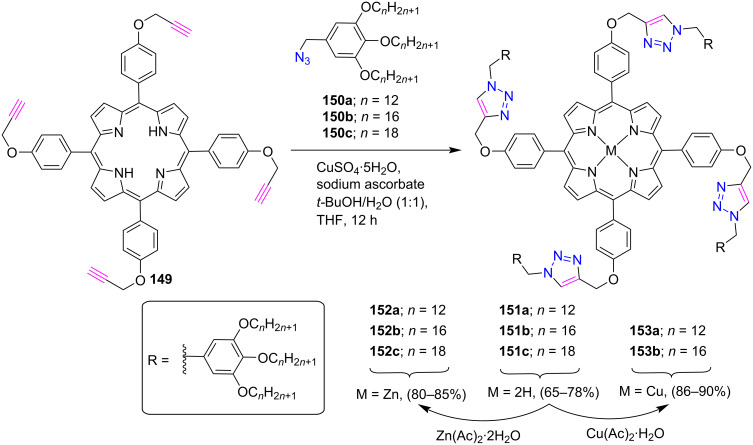
Synthesis of *meso*-tetratriazole-linked porphyrin conjugates **151–153**.

The research group of Cheng [63] designed a click inspired synthesis of novel discotic mesogen bearing a porphyrin unit, a triazole linkage, and a peripheral 3,4,5-trialkoxybenzyl unit (Scheme 32). Firstly, free-base porphyrin conjugates **151a–c** were obtained by the CuAAC reaction between metal free tetraalkynylporphyrin **149** and benzyl azides **150a–c**. Furthermore, the metal-porphyrin conjugates **152a–c** and **153a**,**b** were obtained in good yields from the corresponding free-base porphyrins **151a–c** by the reaction with zinc acetate and copper acetate, respectively. The authors revealed that these compounds can self-assemble into hexagonal columnar phases in their pure states and can form organogels in 1,4-dioxane with an unusual flower-like sphere morphology.

**Scheme 33:**
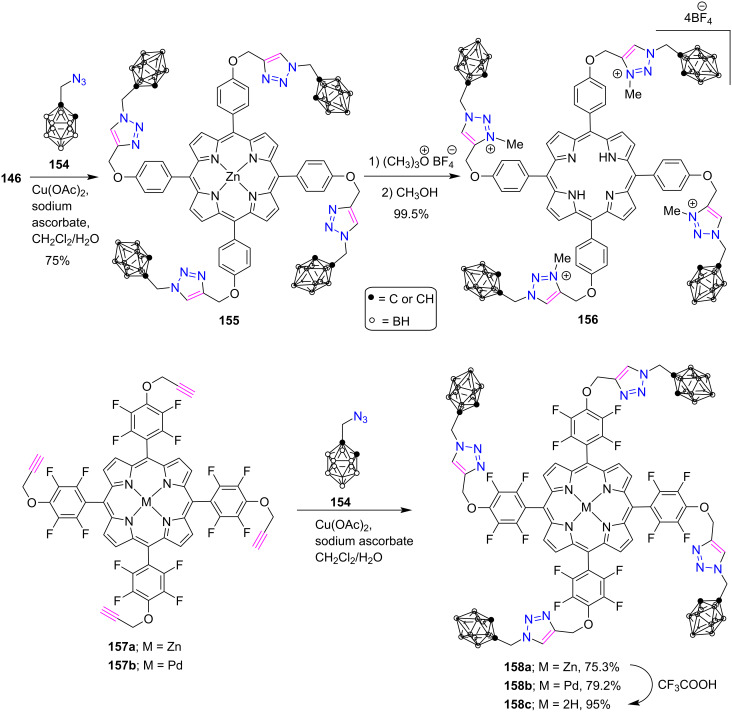
Synthesis of *meso*-tetratrizole-porphyrin**-**carborane conjugates **155**, **156** and **158a**–**c**.

Ol'shevskaya et al. [64] recently established a CuAAC-based synthesis of a novel series of *meso*-triazoloporphyrin-carborane conjugates, as shown in Scheme 33. First, the metalated triazole-linked porphyrin-carborane dyads **155** and **158a**,**b** were synthesized in 75–79.2% yields by the click reaction between porphyrins **146** or **157a**,**b** and azidomethyl-*o*-carborane **154**. Further, porphyrin **155** was alkylated using excess trimethyloxonium tetrafluoroborate in CH_2_Cl_2_ to afford the free-base tetracationic triazolium salt **156** in quantitative yield. Also, a free-base dyad **158c** was obtained in 95% yield by the demetallation of zinc porphyrin **158a** with trifluoroacetic acid in a CHCl_3_/MeOH mixture. These synthesized conjugates showed great affinity for major biological carriers like albumin and LDL. Furthermore, the Zn and Pd porphyrin complexes revealed a good capability to yield singlet oxygen (quantum yield >70%) and exhibited significant photoinduced cytotoxicity.

**Scheme 34:**
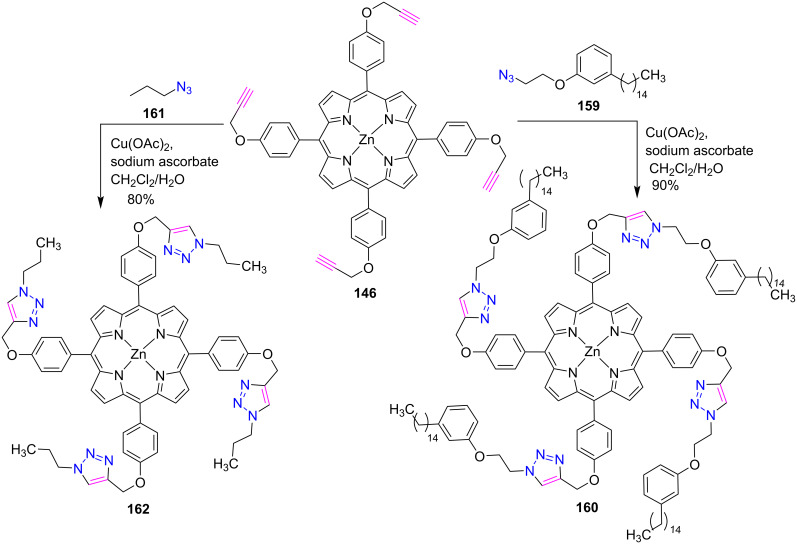
Synthesis of *meso*-tetratriazole-porphyrin-cardanol conjugates **160** and **162**.

In another report, Prakash Rao et al. [65] highlighted the click protocol to accomplish the synthesis of hydrogenated cardanol-triazole-zinc-porphyrin conjugate **160** and triazole-zinc-porphyrin conjugate **162** by the reaction between zinc porphyrin **146** with cardanol azide **159** or **161**, respectively (Scheme 34). Different solvents were used to conduct their UV–vis and fluorescence experiments and the results were compared to other related molecules. Based on the collected data, the porphyrin complex **160** was found to show J-type aggregation in both polar and nonpolar solvents. In polar solvents, aggregation was induced by van der Waals interactions between H-cardanol moieties, while π–π stacking interactions between porphyrin groups were observed in nonpolar solvents. In addition, UV–vis and fluorescence spectra also showed that the 1,2,3-triazole moiety can stabilize zinc porphyrin by forming an intermolecular coordination complex. Also, it was found that porphyrin complex **160** is easy to synthesize, is fat soluble and showed good fluorescence emission; thus, it could be used as a photodynamic therapy application.

**Scheme 35:**
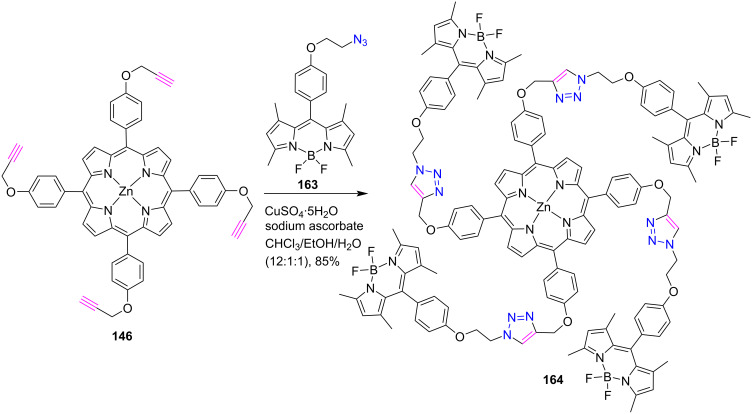
Synthesis of *meso*-tetratriazole-linked porphyrin-BODIPY conjugate **164**.

In 2013, Liu and colleagues [66] used a click chemistry approach to synthesize *meso*-tetratriazole-bridged Zn(II) porphyrin-boron dipyrromethane conjugates **164** in 85% yield by reacting porphyrin **146** with BODIPY **163** containing an azide moiety (Scheme 35). Photophysical studies confirmed that a good spectral overlap was found between the energy donor (BODIPY) and the energy acceptor (porphyrin subunits). Also, complex **164** exhibited a highly efficient photoinduced energy transfer process with an energy transfer quantum yield of 0.98. These conjugates could be useful in light-harvesting applications because of their optical characteristics.

**Scheme 36:**
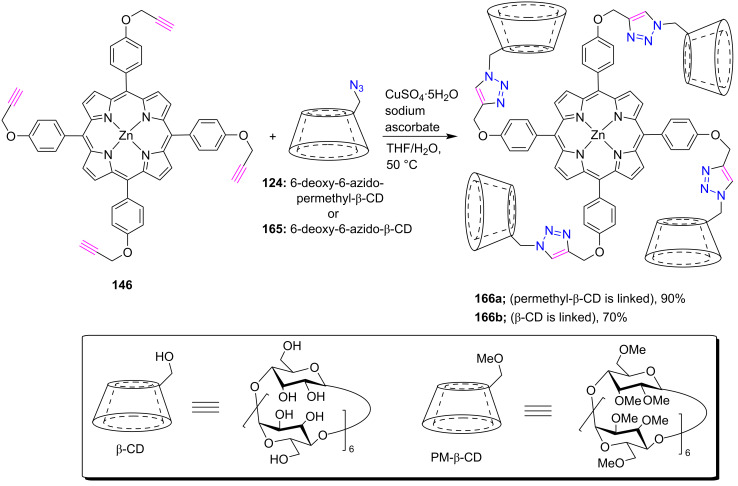
Synthesis of *meso*-tetratriazole-linked porphyrin-β-CD conjugates **166a**,**b**.

In 2008, Liu et al. [67] nicely demonstrated Cu(I)-catalyzed click chemistry for the synthesis of tetrakis(PM-β-CD)-modified zinc(II) porphyrin **166a** and tetra(β-CD)-modified zinc(II) porphyrin **166b** in 90% and 70% yield, respectively (Scheme 36). The synthesis of these porphyrins **166a**,**b** was accomplished by the reaction between porphyrin **146** and 6-deoxy-6-azido-PM-β-CD **124** or 6-deoxy-6-azido-β-CD **165** in the presence of CuSO_4_∙5H_2_O and sodium ascorbate in H_2_O/THF. Furthermore, these two structurally similar porphyrins **166a**,**b** were used as hosts for the intermolecular formation of inclusion complexes with tetrasodium tetraphenylporphyrintetrasulfonate **132** in aqueous solution, leading to the constructions of two different nanoarchitectures with alternating porphyrin and cyclodextrin assembly.

**Scheme 37:**
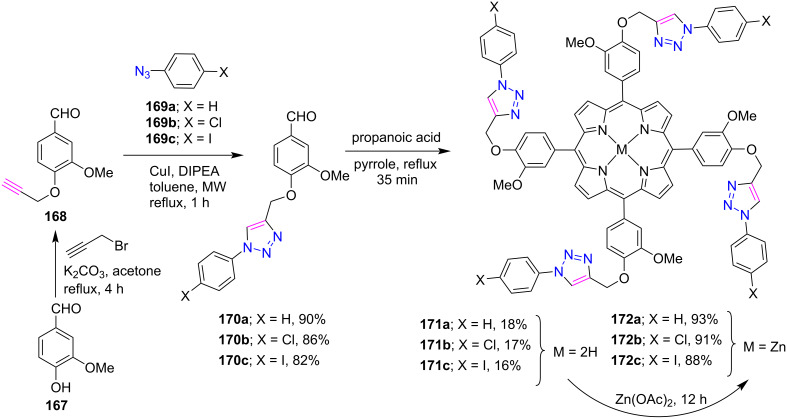
Synthesis of tetratriazole-bridged *meso*-arylporphyrins **171a**–**c** and **172a**–**c**.

Recently, Nasri and co-workers [68] constructed the triazole-bridged *meso*-arylporphyrins **171a**–**c** and **172a**–**c** by using the CuAAC reaction in good yields (Scheme 37). Initially, the triazolo-linked compounds **170a**–**c** were prepared by reacting 3-methoxy-4-(prop-2-ynlyloxy)benzaldehyde (**168**) with azides **169a**–**c** in the presence of CuI and DIPEA in toluene under microwave conditions. Further, three triazole-*meso*-porphyrins **171a**–**c** were prepared using a slightly modified Adler–Longo procedure. Also, the corresponding zinc derivatives **172a**–**c** of these free-base porphyrins were synthesized in excellent yields using Zn(OAc)_2_ in a CHCl_3_/MeOH mixture. Their UV–vis titration study revealed that these host systems exhibit strong anion-binding affinities. Furthermore, with the help of kinetic studies, the absorption efficiency of malachite green dye was investigated by using free-base porphyrins **171a**–**c** and the corresponding zinc(II) compounds **172a**–**c** as adsorbents. In addition to that, various factors affecting the degradation phenomenon have been studied.

#### Octatriazole-bridged porphyrin systems

**Scheme 38:**
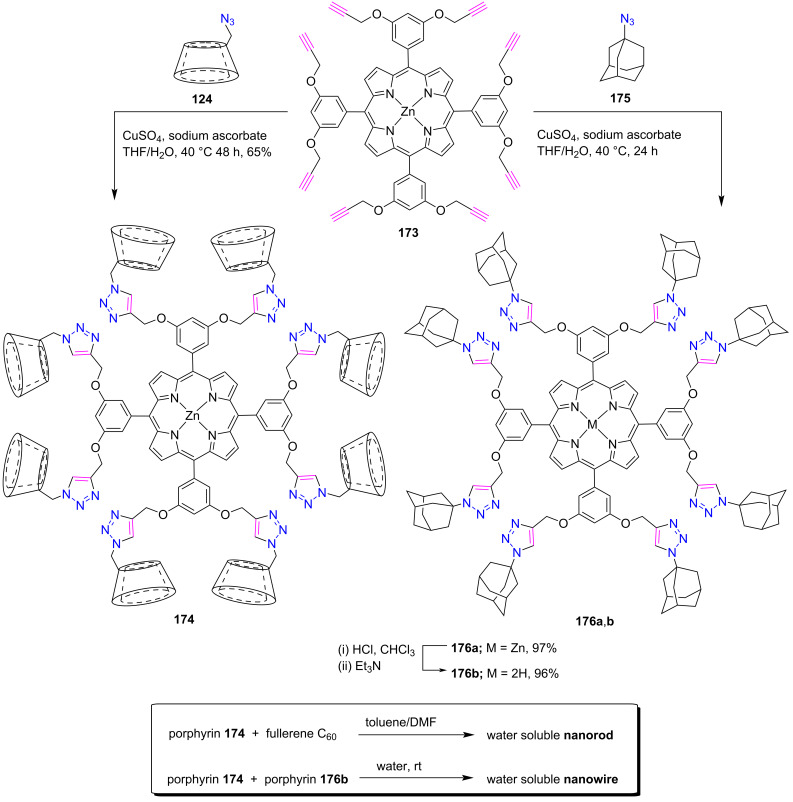
Synthesis of octatriazole-bridged porphyrin-β-CD conjugate **174** and porphyrin-adamantane conjugates **176a**,**b**.

This section describes the preparation of porphyrin conjugates containing eight triazole rings. In 2009, Jayawickramarajah and co-workers [69] synthesized a water-soluble porphyrin-fullerene (C_60_) nanorod through the formation of an inclusion complex between an octa(permethylated-β-CD)-Zn-porphyrin **174** and a pristine C_60_. For the construction of the nanorod, porphyrin **174** was first obtained in 65% yield by a Cu(I)-catalyzed click reaction between 5,10,15,20-tetrakis(3,5-dipropargyloxyphenyl)-Zn-porphyrin **173** and the azide **124** in the presence of CuSO_4_ and sodium ascorbate in THF/H_2_O (Scheme 38). UV–vis spectroscopy showed that the porphyrin **174** was readily soluble in water, and no porphyrin–porphyrin stacking interaction was observed.

In 2010, the same group also reported the construction of a well-defined porphyrin nanowire [70] in water using Zn(II) porphyrin-CD conjugate **174** and free-base porphyrin-adamantane conjugate **176b** through a robust 1:1 β-CD/adamantane host–guest interaction. First, porphyrin **176b** was prepared in 96% by the CuAAC click reaction between octaalkynyl-Zn-porphyrin **173** and 1-azidoadamantane **175**, followed by demetallation with HCl (Scheme 38).

Since porphyrin **174** was water soluble and porphyrin **176b** was insoluble in water; however, when porphyrin **176b** was added to an aqueous solution of porphyrin **174** and stirred at room temperature for 24 h, solubilization occurred, resulting in a homogeneous solution. This is due to the formation of a supramolecular assembly in which the hydrophobic adamantane arms of **176b** are sequestered by the β-CD arms of porphyrin **174**, resulting in water solubility. These prepared porphyrin nanowires were subjected to surface microscopy and fluorescence energy transfer experiments, which elucidated their stability and resistance to disassembly.

## Conclusion

Cu(I)-catalyzed “click chemistry” is a well-established protocol for the facile access of complexes from simple molecular architectures featuring 1,4-disubstituted triazole units with high yield and higher regioselectivity. The click-inspired synthesis and photophysical properties of diverse triazole-linked porphyrin conjugates are discussed in this review. This review includes a number of recent and important research articles in which various chromophoric groups are linked to the porphyrin periphery via an 1,2,3-triazole ring, either at β- or *meso*-positions. First, the review describes the synthesis of β-triazole-linked porphyrins and then *meso*-triazole-linked porphyrins containing one or more triazole rings. The CuAAC click reaction has been shown to bind a variety of chromophores bearing azide or alkyne groups to the porphyrin periphery, including coumarin, xanthone, DNA, ferrocene, corrole, fluorescein, carborane, BODIPY, graphene, carboline, fullerene, NDI, β-cyclodextrin, cholic acid, quinolone, etc. Some of these porphyrin complexes have shown significant electron and/or energy transfer between the porphyrin and the accompanying chromophore, and the 1,2,3-triazole bridge affects charge-separation and photoinduced electron transfer processes between connecting molecules. Various combinations of copper catalysts have been used for the CuAAC-based synthesis of these porphyrin conjugates, the majority of the reports using CuSO_4_ with either ascorbic acid or sodium ascorbate, and Cu(OAc)_2_ with sodium ascorbate under an organic solvent or a mixture of organic solvents and water. In addition, other Cu(I)-catalysts such as CuI, (SIMes)CuBr, Cu(MeCN)_4_PF_6_, and CuBr(PPh_3_)_3_ have been also used in some reactions. Furthermore, a few reports describe the use of ligands like DIPEA, TBTA, NMP, Et_3_N, etc. along with copper catalysts to stabilize the Cu(I)-oxidation state and speed up the click reaction. It has been observed that the presence of a porphyrin ring has little influence on the click reaction and it is compatible with almost all kinds of porphyrin derivatives under different reaction conditions. When an azido or alkyne group is attached to an alkyl chain, the click reaction requires milder reaction conditions and gives higher yields of product than directly linked azido or alkynyl porphyrins. Many applications for porphyrin click products have been proposed, including chemical sensing, anion-binding affinity, photovoltaics devices, light-harvesting materials, and in the synthesis of water-soluble nanowires, nanorods, and nanospheres. In addition, these triazoloporphyrins have a wide range of medical applications, including photoinduced cytotoxicity against cancer cells, drug delivery, as phototherapeutic agents, and PDT applications. I believe this review will be useful and encourage researchers around the world to use CuAAC and its modifications in various fields of industrial and scientific research, which can be useful in materials to medical and other applications.

**Table 1:** Synonyms and abbreviations with their explanations.

Abbreviation	Explanation

BiPy	2,2′-bipyridine
BODIPY	4,4-difluoro-4-bora-3a,4a-diaza-*s*-indacene
Bu_4_NF	tetra-*n*-butylammonium fluoride
β-CD	β-cyclodextrin
CuAAC	copper catalyzed azide–alkyne 1,3-dipolar cycloaddition
Cu(MeCN)_4_PF_6_	copper(I) tetrakis(acetonitrile)hexafluorophosphate
DABCO	1,4-diazabicyclo[2.2.2]octane
DFT	density functional theory
DIPEA	*N,N*-diisopropylethylamine
DMF	*N,N*-dimethylformamide
DNA	deoxyribonucleic acid
DSC	differential scanning calorimetry
DSSC	dye-sensitized solar cell
EIS	electrochemical impedance spectroscopy
Fc	ferrocene
LDL	low-density lipoprotein
MALDI–TOF	matrix-assisted laser desorption ionization–time of flight
MW	microwave
NDI	naphthalenediimide
NG	*N*-doped graphene
NMP	*N*-methyl-2-pyrrolidone
PCEs	power conversion efficiencies
PDT	photodynamic therapy
PL	porphyrin lantern
PM-β-CD	per-*O*-methylated-β-cyclodextrin
PPCs	porphyrin-polymer conjugates
POM	polyoxometalate
SEM	scanning electron microscopy
(SIMes)CuBr	*N,N’*-bis(2,4,6-trimethylphenyl)-4,5-dihydroimidazol-2-ylidene
TBAF	tetra*-n*-butylammonium fluoride
TBTA	tris(benzyltriazolylmethyl)amine
TFA	trifluoroacetic acid
TEM	transmission electron microscopy
TMPyP	*meso*-tetrakis(4-*N*-methylpyridyl)porphine
TMS	tetramethylsilane
ZnP	zinc porphyrin
